# Laboratory Diagnosis of SARS-CoV-2 Pneumonia

**DOI:** 10.3390/diagnostics11071270

**Published:** 2021-07-15

**Authors:** Melissa R. Gitman, Maryia V. Shaban, Alberto E. Paniz-Mondolfi, Emilia M. Sordillo

**Affiliations:** 1 Department of Pathology, Molecular and Cell-Based Medicine, Icahn School of Medicine at Mount Sinai, New York, NY 10029, USA; Alberto.Paniz-Mondolfi@mountsinai.org (A.E.P.-M.); Emilia.Sordillo@mountsinai.org (E.M.S.); 2 Emerging Pathogens and Zoonoses Network, Incubadora Venezolana de la Ciencia, Cabudare 3023, Venezuela; mvshaban@gmail.com

**Keywords:** COVID-19, NAAT, RT-PCR, Ct value, RT-LAMP, rapid antigen test, antibody test, point of care testing

## Abstract

The emergence and rapid proliferation of Coronavirus Disease-2019, throughout the past year, has put an unprecedented strain on the global schema of health infrastructure and health economy. The time-sensitive agenda of identifying the virus in humans and delivering a vaccine to the public constituted an effort to flatten the statistical curve of viral spread as it grew exponentially. At the forefront of this effort was an exigency of developing rapid and accurate diagnostic strategies. These have emerged in various forms over the past year—each with strengths and weaknesses. To date, they fall into three categories: (1) those isolating and replicating viral RNA in patient samples from the respiratory tract (Nucleic Acid Amplification Tests; NAATs), (2) those detecting the presence of viral proteins (Rapid Antigen Tests; RATs) and serology-based exams identifying antibodies to the virus in whole blood and serum. The latter vary in their detection of immunoglobulins of known prevalence in early-stage and late-stage infection. With this review, we delineate the categories of testing measures developed to date, analyze the efficacy of collecting patient specimens from diverse regions of the respiratory tract, and present the up and coming technologies which have made pathogen identification easier and more accessible to the public.

## 1. Introduction

The illness now known as Coronavirus Disease-2019, or COVID-19, was first described in mid-December 2019 when the Wuhan health authorities detected a cluster of cases of atypical pneumonia [1]. As Severe Acute Respiratory Syndrome Coronavirus-2 (SARS-CoV-2), the cause of COVID-19, spread globally, the need for rapid, accurate diagnostic testing was recognized. In this review, we discuss the direct and indirect methods that are currently employed for diagnosis of SARS-CoV-2 infection. Although this virus is relatively new, a plethora of publications have appeared in the last year, and a comprehensive review of all available data is beyond the scope of this paper. We present a brief overview of the virus and available testing options.

## 2. Viral Structure

**Figure 1:**
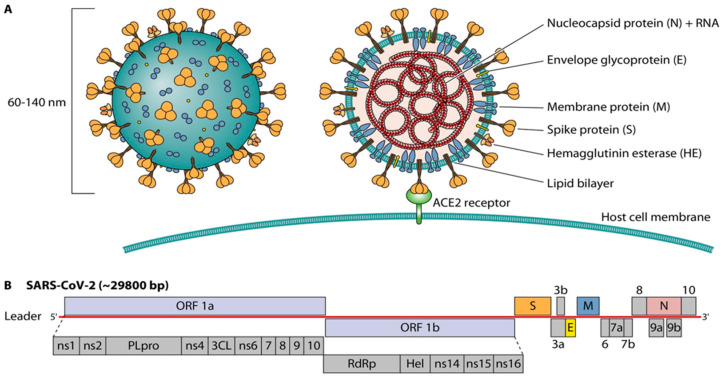
Structure of SARS-CoV-2 virus. A—Schematic of SARS-CoV-2 virion, B—Schematic of SARS-CoV-2 genome structure. Reprinted with permission from ref. [8]. Copyright 2021 American Society for Microbiology-Journals.

Coronaviruses are enveloped, positive-sense, single-stranded RNA viruses [2]. A part of the Coronaviridae family, Betacoronavirus genus, SARS-CoV-2 is the seventh coronavirus known to infect humans [3]. Understanding the structure and genomic architecture of the virus is important, as this is the basis of the targets for the various diagnostic tests. The SARS-CoV-2 virion is roughly spherical and 60–140 nm in diameter [4]. A viral membrane contains the spike (S) glycoprotein, giving the virus its characteristic corona or crown-like appearance [5]. The spike protein features two functional subunits [6]: S1, containing the receptor-binding domain (RBD) that mediates binding to the host cell surface receptor angiotensin-converting enzyme-2 (ACE-2), and S2, which is integral to the subsequent fusion between the viral and host cellular membranes [5]. Other structural proteins include the membrane (M) protein and envelope (E) protein, which create the ring-like structure, and the nucleocapsid (N) protein, which plays a role in successful host cell entry (Figure 1). Additionally, the N-protein is complexed to the single-strand RNA genome, approximately 30 kb in length [7]. The SARS-CoV-2 genome encodes proteases and an RNA-dependent RNA polymerase (RdRp) [6]. The 5′ terminus of the genome contains ORF1ab, which is the largest of all genes [6]. The 3′ terminus contains four structural proteins, S, E, M, N, and eight accessory proteins [6]. A diversity of targets are employed by different test manufacturers, mainly encompassing regions located in the open reading frame (*ORF1*), envelope (*env*), nucleocapsid (*N*), spike (*S*) and RNA-dependent RNA polymerase (*RdRp*) genes. However, mutations across these regions may impact diagnostic performance by affecting specific oligo-binding sites and affecting test sensitivity. Even though SARS-CoV-2 possesses proofreading capacity which makes transcription and replication less prone to mutations, mutational events still occur. Thus, continuous genomic monitoring and target (primer/probe) optimization is key for diagnostic performance.

## 3. Whom to Test

Throughout the pandemic, the populations who meet criteria for testing have evolved as the testing capacity has expanded. Initially, only patients with symptoms compatible with COVID-19 who had traveled to Wuhan, China, were eligible to be tested. As the pandemic progressed and local community transmission was recognized, rapid diagnosis of potentially SARS-CoV-2-infected individuals became crucial in order to cut off chains of transmission, and the requirement for travel was eliminated. As more diagnostic assays became available, screening of potentially exposed but asymptomatic patients became more widespread. Screening in facilities such as nursing homes and other communal living settings has become an indication for testing [9]. Other groups for whom screening of asymptomatic individuals has now been implemented include schools, travelers, healthcare workers, and those with potential exposure to individuals diagnosed with COVID-19 [10]. The Infectious Diseases Society of America (IDSA) has designed an algorithm to assist in the decision of whom to test that stresses the importance of testing symptomatic individuals and lists others who may meet criteria such as recently exposed or pre-procedural and testing available [11].

## 4. What Specimen to Collect

Nasopharyngeal (NP) swabs are the preferred specimen for direct detection of SARS-CoV-2 according to both the World Health Organization (WHO) and the Centers for Disease Control and Prevention (CDC) guidelines.

However, several significant barriers are associated with collection of NP swab specimens for SARS-CoV-2 detection, including the requirement for collection by a trained provider, the use of personal protective equipment (PPE) due to triggering of reflex cough/sneeze during collection, patient discomfort, and potential harm, including a case of a Cerebral Spinal Fluid (CSF) leak due to improper collection [11,12]. Additionally, supply chain disruption resulting in scarcity of the flocked swabs and transport media used for collection of NP specimens has been a concern, particularly during the pandemic surge. As a result, alternate upper respiratory tract specimen types have been evaluated such as anterior nares or nasal swabs. Pere et al. compared NP swabs and nasal swabs in 44 consecutive patients. They detected four false negatives in the nasal swab group (9.1%) and there were no additional positives found in the nasal swab group not detected in the NP swab group [13].

Saliva is another alternative specimen type that has been considered for SARS-CoV-2 diagnostic testing. Advantages of this specimen type include ease of collection, minimal patient discomfort, and improved safety for both patients and providers. As collection of saliva does not provoke a cough/sneeze reflex, guidelines do not require special provider PPE for aerosol exposure. Furthermore, since flocked collection swabs and transport media are not required, reliance on the material supply chain is minimized. In an initial proof of concept, To et al. collected saliva from known positive patients and demonstrated that SARS-CoV-2 could be detected in specimens collected from 11 of the 12 tested [14]. Wyllie et al. compared paired samples of saliva and NP swabs from 70 in-patients with a known diagnosis of COVID-19. At days 1–5 after diagnosis, more patients tested positive from saliva (81%) than NP specimens. (71%). Overall, viral load was higher in saliva than in the paired NP swab specimens (5.58 vs. 4.93 mean log copies per mL) [15]. Conversely, a study of paired NP and saliva samples from 91 patients found NP swab specimens to be slightly more sensitive in the first week and 20% more sensitive if collected in the second week of illness or later [16]. The suitability of saliva for detection of SARS-CoV-2 may reflect overall viral dynamics, since both upper respiratory tract contents as well as lower respiratory secretions driven by the mucociliary airway epithelium combine in the oral cavity. Of note, a recent study from Huang et al. demonstrated higher RNA expression of the ACE2 receptor and the TMPRSS2 internalization protease in epithelial cells of the glands and oral mucosae, which translated into a higher salivary viral burden [17]. Moreover, recent evidence provided by Savela et al. suggests that high-sensitivity saliva-based tests can detect the presence of SARS-CoV-2 earlier in the infection when compared to self-collected anterior-nares nasal swabs [18].

SARS-CoV-2 can also be detected in lower respiratory specimens, although reports from different investigators are contradictory regarding diagnostic sensitivity, perhaps influenced by stage of disease or other patient characteristics. Possible lower tract specimens include sputum, tracheal aspirates and bronchoalveolar lavage (BAL). Tracheal aspirates are much simpler to obtain in intubated patients; however, unlike BAL fluid, the viscosity of the specimens vary. However, BAL has the disadvantage of requiring an invasive procedure. Some studies have suggested that lower respiratory tract specimens may be persistently positive longer than upper tract specimens. In a case reported from Thailand, a patient whose NP and oropharyngeal swab specimens had twice tested negative was diagnosed by RT-PCR detection of SARS-CoV-2 in a bronchoalveolar lavage (BAL) specimen collected on hospital day 8 [19]. One potential explanation for this is that lower respiratory tract epithelial cells and pneumocytes have a higher density of ACE2 receptors that serve as the target for SARS-CoV-2 compared to upper tract epithelial cells [20,21].

Understanding SARS-CoV-2 viral dynamics in the respiratory system may help to better guide specimen collection. However, studies addressing SARS-CoV-2 tissue distribution and dynamics are still scarce. Viral loads are known to differ amongst sample types, being higher in respiratory samples at early stages of the disease. For example, a meta-analysis comparing 3442 respiratory samples, including 1299 nasopharyngeal swabs, 1083 oropharyngeal swabs and 1060 sputum samples, indicated that sputum (71% positive samples) was far more sensitive than oropharyngeal swabs (54%) or nasopharyngeal swabs (43%), although when stratified by time since onset of symptoms, sensitivity was diminished for all three specimens types the later in the disease course the specimens were collected [22]. Interestingly, Hou et al. utilized reverse genetics to show a variable infectious gradient throughout the respiratory tract that parallels tissue expression of the ACE-2 receptor [23]. In addition, they assessed viral tropism and distribution in lungs from deceased COVID-19 patients, demonstrating a higher gradient of virus in the upper respiratory tract vs. the lower tract, and suggested that infection of type 2 pneumocytes in the alveolar region is seeded by aspiration of the virus in upper airway secretions [23]. A separate meta-analysis comparing different methodologies identified that pooled nasal and throat swabs gave the highest sensitivity of 97%, followed by nasal swabs (86%) and saliva (85%) and finally throat swabs alone (68%). Moreover, in a different meta-analysis by Weiss et al., it was found that SARS-CoV-2 PCRs remained positive longer in the lower respiratory tract (LRT) compared to the upper respiratory tract (URT), both in mild disease (5.7 days) and moderate–severe disease (5.9 days). In patients with mild disease, the viral load peaked on day 4 of illness with a maximum viral load of 6.61 × 108 viral copies/mL in the URT, as compared to the LRT, which peaked on day 6 at 2.69 × 108 copies/mL. In patients with moderate to severe disease, the URT viral load peaked at 4.60 × 109 copies/mL on day 8 as compared to the LRT with a peak of 3.45 × 108 copies/mL on day 11.

Once the site of sample collection has been determined, the next consideration is the collection device and transport conditions. Traditionally, samples collected for viral testing are transported in viral transport media (VTM), which contains a mix of Hanks balance salt solution, antibiotics, antifungals, and a PH indicator. At the onset of the pandemic, these were in short supply. Many centers tested using saline or phosphate buffered saline (PBS) as transport media and found these performed as well as VTM [24,25]. It is recommended that samples be stored at 2–80 °C and tested within 72 h, otherwise they should be frozen until they can be tested [10] in order to preserve nucleic acid stability and integrity.

## 5. Types of Diagnostic Testing

### 5.1. Nucleic Acid Amplification Tests (NAAT)

Detection of the presence of viral RNA using NAAT has become the mainstay of SARS-CoV-2 diagnosis with real-time polymerase chain reaction (RT-PCR) being the most common NAAT method currently employed [10]. RT-PCR involves several steps, the first being extraction of viral RNA from the collected specimens using silica or magnetic particle-based methods. Next, there is reverse transcription of the viral RNA to a single-stranded DNA copy (cDNA) using a reverse transcriptase enzyme. Finally, there is the amplification step where the extracted viral RNA is mixed with reagents containing the primers, probes and master mix and detection is obtained through a coupled fluorescent marker [26]. The first RT-qPCR for SARS-CoV-2 detection was developed by China’s Center for Disease Control and Prevention and was designed to detect the N and the ORF1ab genes [27]. Next, the Charite Institute developed a protocol incorporating primers targeting genes of the E, N, and RdRp [27]. In the U.S., the first RT-PCR protocol was developed by the CDC and targeted N1 and N2 [27]. Internationally, there are many different primers and probe sets available from the World Health Organization [28]. Vogels et al. compared seven primer sets and found they all had similar performance characteristics [29]. It is important to mention that, as specified by Corman et al., the performance varies for different targets across the viral genome, potentially affecting sensitivity and specificity [30].

In the U.S., multiple commercial assays are now marketed [31] and several assays are available as part of a test system that utilizes a single, self-contained, high-throughput instrument to perform all the PCR or other NAAT steps (Table 1). To address the urgent need for diagnostic test capacity, these tests were reviewed by the US Food and Drug Administration (FDA) through an emergency use authorization pathway. The early EUA process required companies to submit analytic sensitivity, limit of detection, along with cross-reactivity studies [32]. Initial studies submitted to the FDA were performed using contrived samples, not patient samples, and therefore clinical correlation was not performed. Consequently, determination of sensitivity and specificity against a clinical gold standard is not generally available for these assays [31].

Assay selection relies on several factors, including availability of reagents, cost, throughput and performance characteristics [32]. Various publications have appeared over the past year, comparing the performance characteristics of the different commercial assays. One publication from Moran et al. tested 103 specimens on the Roche cobas SARS-CoV-2 test and Cepheid Xpert Xpress SARS-CoV-2 assay and found that 42 tested positive and 60 tested negative with both systems for agreement of 99% [60]. Rhoads et al. tested 96 samples using the CDC EUA as the gold standard and found the positive predictive value of the Diasorin Simplexa COVID-19 Direct assay to be 96% with four false negative samples [53]. A multicenter study of the Cepheid Xpert Xpress SARS-CoV-2 test, a rapid PCR instrument widely available in many clinical labs, demonstrated an LOD of 0.01 PFU/mL. When residual clinical samples were compared to various other PCR assays, it demonstrated a 99.5% positive agreement and 95.8% negative agreement [33]. Fung et al. compared the analytic limit of detection across seven molecular platforms using a pool of positive patient samples quantified via digital droplet PCR. Limits of detection ranged from ≤10 to 74 copies/mL for commercial high-throughput laboratory analyzers (Roche cobas SARS-CoV-2 Test, Abbott RealTime SARS-C0V-2 assay and Hologic Panther Fusion SARS-CoV-2 assay) and 167 to 511 copies/mL for sample-to-answer (DiaSorin Simplexa COVID-19 Direct assay, GenMark ePlex SARS-CoV-2 Test) and point-of-care instruments (Abbott ID NOW COVID-19 test) [34]. Overall, most commercial RT-PCR assays show excellent analytic sensitivity [38,61,62,63,64,65,66,67,68]. Differences in results may be due to a number of factors, including whether there is a full extraction step prior to amplification [69], whether the patient has a low viral load close to the limit of detection of the assay [69], volume of sample tested and assay design, such as targets chosen [32]. Finally, the quality of the sample received likely has a significant impact on the likelihood of a reliable result [32,70].

One potential advantage of RT-PCR-based tests is the ability to generate a cycle threshold (Ct) value. Ct values have been suggested as a surrogate for viral load [71,72]. The Ct value refers to the number of PCR cycles needed to amplify the target sequence to a detectable level [26]. A specimen containing more virus (higher viral load) will need fewer PCR cycles to produce a positive signal; thus, a lower Ct value implies a higher viral load in the original sample. In assays with more than one target, more than one Ct value may be generated. However, caution must be used in the interpretation of Ct values generated by assays intended to produce a qualitative result (e.g., detected or positive vs. not detected or negative) and that have not been designed to produce quantitative results or and have not been standardized against control samples of a known concentration. In some NAAT assays, the relationship between the Ct vale and the concentration of target RNA in the sample may not be linear [72]. Another caveat is that a Ct value generated in one assay platform cannot be correlated directly with a value generated using a different assay due to heterogeneity among the targets and amplification protocols used by different assays and test platforms. Additionally, variability in collection techniques as well as the intrinsic heterogeneity of specimens that contain respiratory secretions contribute to the difficulty of standardizing the viral loads in these samples [72]. Further complicating interpretation of their significance is the finding that Ct values overlap in specimens from symptomatic and asymptomatic patients [73,74] and may not always indicate disease severity.

Disadvantages of PCR testing include the need for specialized and costly reagents, expensive laboratory instrumentation, and highly skilled laboratory personnel. Processing large numbers of specimens is time-consuming and even with automation of many processing steps can take a prolonged period of time to generate results [75,76]. One major limitation of RT-PCR methods is that the tests are overly sensitive and do not distinguish between active infection and non-viable virus [76]. It has been demonstrated that in individuals with COVID-19, viral shedding approximately begins 2–3 days before symptoms appear [77]. The average duration of viral shedding is 20 days [78]; however, viral shedding by immunocompromised hosts may be prolonged for 3–5 months or more [79,80]. In some cases, the shed virus may still be viable. Shedding of replication-competent SARS-CoV-2 has been shown in cell cultures inoculated with respiratory samples from two patients with prolonged, severe COVID-19 [81].

Transcription-mediated amplification is an alternate type of NAAT testing that forms the basis for the APTIMA assay performed on the high-throughput Panther platform. After hybridizing the viral RNA to a T7 promoter primer, it is reverse-transcribed into a complementary cDNA. The target RNA strand is subsequently degraded by RNAse H, leaving a single-stranded cDNA that includes the T7 promoter. An additional primer is used to generate a double-stranded DNA. T7 RNA polymerase then transcribes the ds-DNA into RNA amplicons, which can then restart the process, allowing this exponential amplification [82]. Its performance characteristics suggest the assay is highly sensitive [83].

Other NAAT methods that have been considered for SARS-CoV-2 test manufacturing include reverse transcriptase loop-mediated isothermal amplification (RT-LAMP), nicking endonuclease amplification reaction (NEAR), and Recombinase Polymerase Amplification (RPA) [84]. The increasing need for efficient and rapid testing has also opened the door to alternate approaches such as the quantitative Selective Temperature Amplification Reaction (qSTAR) technology, a qualitative non-isothermal nucleic acid amplification technique based on a two-step cycling protocol that capitalizes on the activity of polymerase and a nicking enzyme and shuttling between temperatures [85]. For RNA STAR, complete incorporation of an extraction buffer allows the sample to be loaded directly, removing the extraction step and significantly shortening the hands-on time by combining lysis and amplification into a single step [85].

Finally, there has been much interest in use of innovative technologies such as clustered regularly interspaced short palindromic repeats (CRISPR) [48], Specific High Sensitivity Enzymatic Reporter UnLocking (SHERLOCK) [56] and MassARRAY [86]. The MassARRAY (Agena Bioscience) is a novel multiplex reverse transcription RT-PCR/MALDI-TOF-based system with a scalable high-throughput capacity and an increased sensitivity achieved by coupling of RT-PCR and mass spectrometry along with a multitarget (N1, N2, N3, ORF1ab, orf1) interrogation of the viral genome. However, a possible limitation to the use of platform as an end-point detection method is that it relies on end-product quantification, and thus does not generate Ct values. To date, the MassARRAY has obtained an FDA EUA for SARS-CoV-2 detection in upper respiratory (nasopharyngeal swab, oropharyngeal swab, nasal and mid-turbinate swabs, and nasal and nasopharyngeal aspirate) and lower respiratory (bronchoalveolar lavage) specimens collected and stored in VTM or UTM [87].

SARS-CoV-2 genetic variability has become a pressing issue, particularly in the context of the worldwide emergence of variants of concern and the potential to affect diagnostic performance. Of note, early in the pandemic, Artesi et al. reported on a recurrent mutation at position 26,340 of SARS-CoV-2 associated with dropout of the E gene on the Roche cobas SARS-CoV-2 Test [88]. More recently, Bal et al. reported a spike deletion H69-V70 identified in several variants, leading to S gene target dropout in the TaqPath qPCR (Thermo Fisher, Waltham, MA, USA) assay [43]. A recent study by Wang et al. using a nucleotide- and gene-based analysis determined that of all currently targeted genes, the N gene is the most prone to mutations, followed by the E gene, as opposed to ORF1ab, which showed a higher diagnostic reliability [89].

Even though the impact of variants on test performance is currently predicted to be low, laboratories should monitor routinely for target failure. This also highlights the importance of incorporating redundancy by targeting more than one target of the viral genome in order to decrease the likelihood of false negative results.

The need for rapid, accurate diagnosis to facilitate appropriate implementation of respiratory isolation measures and patient management has led to the development of point of care testing (POCT); however, there have been concerns with the performance of these tests in some clinical settings. There are currently six rapid point-of-care (POC) NAATs that have received emergency use authorization (EUA) by US Food and Drug Administration (FDA) for point of care use to detect SARS-CoV-2: the Cue COVID-19 test, Abbott ID NOW, Cepheid Xpert Xpress SARS-CoV-2 test, Roche Cobas SARS-CoV-2 & Influenza A/B on the Cobas Liat System, Mesa BioTech Accula SARS-CoV-2 and BioFire Respiratory Panel 2.1-EZ.

Hansen et al. compared results from 357 nasopharyngeal swabs tested on the cobas SARS-CoV-2 & Influenza A/B Assay run on the Laboratory in a tube (LIAT) instrument to the Roche cobas SARS-CoV-2 Test. The overall agreement was found to be 98.6% (352/357); the positive percent agreement for SARS-CoV-2 was 100% (162/162) and the negative percent agreement was 97.4% (190/195) [36]. Although overall this study demonstrated excellent performance characteristics, the FDA recently issued a warning concerning the risks for false positives [90]. Another common POCT test, the Abbott ID now COVID 19 test, was compared to the Cepheid Xpert Xpress SARS-CoV-2 assay using dry nasal swabs. Overall, results from testing by the ID Now agreed for 17 of 31 positive Xpert Xpress samples, with a positive percent agreement of 54.8%. The ID Now results matched 69 of the 70 negative Xpert Xpress results, with 1 positive detected by ID Now but not by Xpert Xpress, for a negative percent agreement of 98.6% [91]. Multiple studies have found lower positive agreement for the ID NOW in comparison to more sensitive molecular platforms [54,60,61,63]. As there is a need for rapid testing results in many settings, particularly emergency rooms, it has been proposed that multistep testing algorithms be developed [92]; however, further research is likely required to formalize this guidance. Another point of care NAAT test is the Cue COVID-19 Test for Home and Over the Counter (OTC) Use. It is an isothermal nucleic acid amplification assay which tests anterior nares samples that are self-collected by adults or collected by an adult from children. One study from the Mayo clinic compared results from the Cue POCT to standard of care testing in a central lab using either the Hologic assay or an LDT [93]. They found excellent positive and negative predictive values; however, of note, in this study, the Cue swabs were collected by nurses and the testing was carried out by MLTs, not patients, as the product is designed for [93].

### 5.2. Rapid Antigen Tests (RATs)

Rapid antigen tests (RATs), which detect the presence of viral proteins, are another form of POCT (Table 2). Most of these types of tests are lateral-flow immunoassays that create a fluorescent band on the test strip if the target viral proteins are present [6]. These tests are usually cheap and rapid. Beck et al. compared 346 paired NPS tested on both the Sofia Quidel and Hologic Panther Aptima assays and found overall lower agreement amongst the positive samples compared to the negatives (PPA 77% vs. NPA 99.6%). However, it is notable that the PPA of the SOFIA test with the APTIMA TMA test was 82.0% for patients tested ≤ 5 days from symptom onset and 54.5% for patients tested > 5 days from symptom onset [94]. Young et al. compared the BD Veritor System for Rapid Detection of SARS-CoV-2 to the Lyra SARS-CoV-2 assay and, in a separate analysis, compared the BD Veritor System for Rapid Detection of SARS-CoV2 antigen test to the Sofia SARS Antigen FIA. Compared to the Lyra PCR assay, the positive predictive agreement was 88% for patients with two or more symptoms consistent with COVID-19 but dropped to 57.1% and to 66.7% for specimens from patients with only one symptom. Overall, the two rapid antigen tests were reported to have similar performance characteristics [95]. A Cochrane review of rapid antigen tests found that test sensitivity ranged from 30% to 80% with a pooled sensitivity of 56% [96]. It has been proposed that despite a lower positive predictive value in comparison to NAAT tests, there are potential uses for antigen tests. One use could be for rapid diagnosis of symptomatic patients. However, negative results would not rule out asymptomatic carriage and would likely still require PCR confirmation, and positive findings in asymptomatic require a second methodology to rule out a false positive result. Another potential use for the test might be to predict which patients are likely to be infectious to others. One group demonstrated that antigen positivity has a 90% PPV of having culturable virus vs. only 70% amongst PCR positive patients [97]. The CDC has proposed an algorithm for how to best use antigen testing [98]. Even though most of the commercially available antigen tests target the viral nucleocapsid protein (N gene), the very few assays that target the spike protein (S gene) may be negligibly affected by current emergent variants. However, to date, there are no studies confirming the potential impact of variants in antigen-based test performance [99].

### 5.3. SARS CoV-2 Antibody Tests

Serologic testing is an important tool at the population level, since establishing seroprevalence can enhance understanding of the epidemiology of COVID-19 and assist in public health planning. For an individual, serology can help establish potential susceptibility to infection or may be used as an adjunct to PCR testing for diagnosis, particularly when patients present late in the course of illness when the virus may no longer be detectable [107]. For symptomatic individuals, IgM can be detected at a median of 5 days, and IgG at a median of 14 days, after symptom onset [108]. IgG antibodies have been shown to correlate with disease severity, decline at varying rates, and may be detectable for months following infection [109,110]. However, between 4% and 10% of the individuals with confirmed SARS-CoV-2 infection may have an undetectable or delayed antibody response [111]. The humoral response includes antibodies directed against RBD, S and N proteins, which serve as the primary targets for COVID-19 serologic assays [6]. Some general limitations of serology studies for COVID-19 include: it is unknown how long protection lasts; protection is unlikely to be durable given short-lived immunity to other coronaviruses; some people with mild/asymptomatic infections never develop antibody responses at all; serology cannot be used in acute diagnosis; combination IgM/IgG assays are of questionable value due to concerns of cross-reactivity of IgM and other coronavirus antibodies [6,82,107,112,113].

At present, serology is not recommended to assess the response to COVID-19 vaccination. Since vaccines induce antibodies to specific viral protein targets, post-vaccination serologic test results will be negative in persons if the test used does not detect antibodies induced by the vaccine [113]. As for direct viral testing, there is no gold standard for antibody testing. RT-PCR positivity is currently used as a surrogate; however, patients may have negative serologic results with a positive PCR test for any number of reasons including: (1) failure to mount a measurable serologic response in immunocompromised hosts; (2) clearance of infection by T cells or other immune mechanisms; (3) presence of an interfering substance in the patient’s serum; (4) false positive PCR result in a patient who did not have an infection with SARS-CoV-2 [114].

Serologic testing assays can be broadly divided into three main categories: detection of total antibodies, detection of specific antibody subclasses (IgG, IgM or IgA), and detection of neutralizing antibodies (nAbs) using qualitative or semi-quantitative methods [107]. Detection of IgA is not routinely recommended for clinical assays [113]; detection of total antibodies may enhance sensitivity [114]. Currently, commercially produced antibody methods include rapid diagnostic tests (RDTs), enzyme-linked immunosorbent assays (ELISA) and chemiluminescence immunoassays (CLIAs) [107]. RDTs are based on antibody detection using a nitrocellulose membrane. The sample is loaded onto a loading pad and then flows via capillary motion when buffer is added. Antibodies in the specimen bind to nanoparticles on the membrane which then are captured by anti-human antibodies. The convenience of these assays makes them popular as POCTs but there has been a wide variability in performance characteristics for these assays across different vendors. For ELISA-based methods, patient specimens are added to a tube or well of a multiassay plate coated with viral antigens. A secondary (anti-human) antibody is linked to an enzyme that will produce a color readout when its substrate is added. Development of color when the substrate is added indicates that a human antibody to the viral antigen bound to the well or tube was present in the patient specimen. Multiwell plate assays or high-throughput platforms allow for large-scale testing but are expensive and must be performed in a laboratory. CLIA is a similar technique to ELISA, but a fluorescent molecule is used as the indicator [107].

Several available serologic assays were compared directly by the National SARS-CoV-2 serology assay evaluation group, including four commercial and one home-brew serologic assay: the SARS-CoV-2 IgG assay (Abbott), Liaison SARS-CoV-2 S1/S2 IgG assay (Diasorin), Elecsys Anti-SARS-CoV-2 assay (Roche), SARS-CoV-2 total assay (Siemens) and the Oxford Immunoassay. The results for 976 pre-pandemic blood samples and 536 samples from patients with confirmed SARS-CoV-2 infection, collected > 20 days after symptom onset, were evaluated. All five assays demonstrated a sensitivity and specificity of >90%, with a sensitivity of >98% for specimens obtained > 30 days after symptom onset [114]. Similar findings have been obtained in other comparative studies [115,116,117,118]. There are currently over fifty serologic assays with FDA EUA [32]. As a result, comparison of performance can be difficult due to differences in the approach, the sample size, sample collection time and disease prevalence in the population tested by each manufacturer, which vary widely [107]. A summary of some of the currently available serologic tests can be found in Table 3.

The diversity of molecular assays targeting different SARS-CoV-2 genes has enhanced diagnosis capacity and precise identification of SARS-CoV-2 infections. However, the accumulation of nucleotide changes during viral replication has led to the emergence of variant viruses and potentially impacts diagnostic test accuracy for detection of SARS-Cov-2 infection. This highlights the importance of monitoring performance of already released and under-development assays, as well as the need for continued genomic surveillance aimed at detection of variants and their impact on diagnostic target regions. Additionally, the wide circulation of virus variants and concerns regarding the ability of current molecular assays to reliably identify future SARS-CoV-2 emerging variants emphasizes the pressing need for increased and timely whole genome sequencing and surveillance.

## 6. Conclusions

In one year, we have learnt a great deal about SARS-CoV-2, a novel coronavirus. As the pandemic continues, the state of knowledge will continue to evolve. This review provides an overview to assist providers in better understanding the testing options for detection of SARS-CoV-2 infections and to assist in the management of patients with COVID-19.

## Figures and Tables

**Table 1:** Comparative list of nucleic acid amplification tests for the detection of SARS CoV-2, by manufacturer, origin of specimen, target gene, sensitivity, specificity, limit of detection and the time to produce one successful run.

Nucleic Acid Amplification Tests (NAAT)									
Name of Test	Developer	Nature of Specimen	Target Gene	Sensitivity (PPA)	Specificity (NPA)	Limit of Detection (LoD)	Time to Result	Status	References
**Non-Isothermal: RT-PCR**									
Xpert ^®^ Xpress SARS-CoV-2/Flu/RSV	Cepheid	NS, NPS, OPS, MTS, NA, NW	*E*, *N2*	97.9%	100%	131 GCE/mL	45 min run	EUA	[33,34,35]
Cobas ^®^ SARS-CoV-2 & Influenza A/B: cobas Liat System	Roche Molecular Systems	NPS, NS	*Orf1ab*, *N*	100%	97.4%	0.012 TCID_50_/mL	20 min run	EUA	[36,37]
Cobas ^®^ SARS-CoV-2 Test	Roche Molecular Systems	NS, NPS, OPS	*Orf1ab*, *E*	100%	95.5%	0.007 TCID_50_/mL	3.5 h run	EUA; CE-IVD	[33,38,39,40]
Accula ™ SARS-CoV-2 Test	Mesa Biotech	NS, MTS	*N*	95.8%	100%	150 copies/mL	30 min run	EUA	[41]
BioFire ^®^ Respiratory Panel 2.1-EZ	BioFire Diagnostics	NPS	*S*, *M*	98%	100%	6000 copies/mL	45 min run	EUA	[42]
TaqPath ™ COVID-19 Pooling Kit	ThermoFisher Scientific, Inc.	NPS, NA, BAL	*Orf1ab*, *S*, *N*	100%	100%	10 GCE/reaction	4 h run	EUA; CE-IVD	[43,44,45]
Abbott RealTime ™ SARS-CoV-2	Abbott Molecular	NS, NPS, OPS, BAL	*RdRp*, *N*	100%	100%	100 copies/mL	6.8 h run	EUA	[34,46]
Non-Isothermal: RT-qSTAR amplification									
PerkinElmer ^®^ SARS-CoV-2 RT-qPCR Reagent Kit	Perkin Elmer Genomics	NPS, OPS, NA	*Orf1ab*, *N*	100%	100%	120 copies/mL	1 h run	CE-IVD; WHO-EUL	[47]
LumiraDx ™ SARS-CoV-2 RNA STAR complete	LumiraDx UK, Ltd.	NPS, OPS, NA, MTS	*Orf1ab*	95%	100%	1875 copies/mL	20 min run	EUA	[48]
**Isothermal: Transcription mediated amplification**									
Aptima ^®^ SARS-CoV-2 Assay	Hologic, Inc.	NPS, OPS, NS, NA	*Orf1ab*	100%	99.7%	0.026 TCID_50_/mL	2.5 h run	EUA; CE-IVD	[49]
**ISOTHERMAL: RT-LAMP/NEAR**									
Cue ™ COVID-19 Test for Home and OTC Use	Cue Health, Inc.	NS	*N*	97.4%	99.1%	2700 copies/mL	20 min run	EUA	[50]
AQ-TOP ™ COVID-19 Rapid Detection Test PLUS	Seasun Biomaterials, Inc.	NPS, OPS, NS, NA, MTS	*Orf1ab*, *N*	100%	100%	1 copy/µL	2 h run	EUA	[51]
Pro-AmpRT SARS-CoV-2 Test	Pro-Lab Diagnostics	NPS, OPS, NS, NW, MTS	*Orf1ab*	96.6%	100%	125 copies/swab	30 min run	EUA	[52]
ID Now ™ COVID-19 (NEAR)	Abbott Diagnostics	NPS, OPS, NS	*RdRp*	100%	100%	125 GCE/mL	13 min run	EUA; CE-IVD	[53,54,55]
**isothermal: RT-PCR/CRISPR**									
Sherlock ™ CRISPR SARS-CoV-2 Kit	Sherlock Biosciences Inc.	NPS, OPS, NS, NPW, NA, BAL	*Orf1ab*, *N*	100%	100%	6750 copies/mL	1h run	EUA	[56,57]
SARS-CoV-2 DETECTR ™ Reagent Kit	Mammoth Biosciences, Inc.	NPS, OPS, MTS, NPA, NA	*N*	95%	100%	20,000 copies/mL	15 min run	EUA	[58]
Caspr Lyo-CRISPR SARS-CoV-2 Kit (FAM)	Caspr Biotech	NS, NPS, OPS	*Orf1ab*, *N*	99%	99%	25,000 copies/mL	1 h run	EUA	[59]

Abbreviations: PPA, Positive Percentage Agreement; NPA, Negative Percentage Agreement; RT-PCR, Reverse Transcriptase-Polymerase Chain Reaction; RT-qSTAR, Reverse Transcriptase-Selective Temperature Amplification Reaction; RT-LAMP, Reverse Transcriptase-Loop Mediated Isothermal Amplification; NEAR, Nicking Enzyme Amplification Reaction; CRISPR, Clustered Regularly Interspersed Small Palindromic Repeats; NPS, Nasopharyngeal Swab; OPS, Oropharyngeal Swab; NS, Nasal Swab; NPA, Nasopharyngeal Aspirates; NA, Nasal Aspirates; NW, Nasal Wash; MTS, Mid-turbinate Nasal Swab; BAL, Bronchiolar Lavage; *E*, Envelope Protein; *N*, Nucleocapsid Protein; *S*, Spike Protein; *M*, Membrane Protein; TCID_50_, tissue culture infectious dose infecting 50% of cells; GCE, Genomic copy equivalents; EUA, Emergency Use Authorization.

**Table 2:** Comparative list of rapid antigen tests for the detection of SARS CoV-2, by manufacturer, origin of specimen, target gene, sensitivity, specificity, limit of detection, and the time to produce one successful run.

Rapid Antigen Tests (RATs)									
Name of Test	Developer	Nature of Specimen	Target Antigen	Sensitivity (PPA)	Specificity (NPA)	Limit of Detection (LoD)	Time to Result	Status	References
Sofia ^®^ SARS Antigen FIA	Quidel Corporation	NPS, NS, ANS	Nucleocapsid Protein	100%	100%	113 TCID_50_/mL	15 min run	EUA	[94,95,100]
BD Veritor ™ System for RAPID Detection of SARS-CoV-2 & Flu A+B	Becton, Dickinson and Company	NS	Nucleocapsid Protein	84%	100%	140 TCID_50_/mL	15 min run	EUA	[95,101]
InteliSwab ™ COVID-19 Rapid Test Pro	OraSure Technologies, Inc.	NS	Nucleocapsid Protein	84%	98%	2500 TCID_50_/mL	35 min run	EUA: CE-IVD	[102]
SCoV-2 Ag Detect ™ Rapid Test	InBios International, Inc.	ANS, NS	Nucleocapsid Protein	86.6%	100%	6300 TCID_50_/mL	25 min run	EUA; CE-IVD	[103]
Celltrion DiaTrust ™ COVID-19 Ag Rapid Test	Celltrion USA, Inc.	NPS	Nucleocapsid Protein, Spike RBD	93.3%	99%	32 TCID_50_/mL	15 min run	EUA	[104]
BinaxNOW ™ COVID-19 Ag Card2 Home Tool	Abbott Diagnostics Scarborough, Inc.	NS	Nucleocapsid Protein	84.6%	98.5%	140.6 TCID_50_/mL	15 min run	EUA	[105]
Status ™ COVID-19 Antigen Test	Princeton BioMeditech Corporation.	NPS	Nucleocapsid Protein	93.9%	100%	2700 TCID_50_/mL	15 min run	EUA	[106]

Abbreviations: PPA, Positive Percentage Agreement; NPA, Negative Percentage Agreement; NPS, Nasopharyngeal Swab; OPS, Oropharyngeal Swab; ANS, Anterior Nasal Aspirates; NS, Nasal Swab; NPA, Nasopharyngeal Aspirates; NA, Nasal Aspirates; NW, Nasal Wash; MTS, Mid-turbinate Nasal Swab; BAL, Bronchiolar Lavage; TCID_50_, tissue culture infectious dose infecting 50% of cells; GCE, Genomic copy equivalents; EUA, Emergency Use Authorization.

**Table 3:** Comparative list of serology tests for the detection of SARS CoV-2 by manufacturer, origin of specimen, target antibody, sensitivity, specificity and the time to produce one successful run.

Serology Tests								
Name of Test	Developer	Nature of Specimen	Target Antibody	Sensitivity (PPA)	Specificity (NPA)	Time to Result	Status	References
**Rapid Serology Test (RST)**								
MidaSpot ™ COVID-19 Antibody Combo Detection Test	Nirmidas Biotech, Inc.	WB, EDTA P, LHP, S	IgM, IgG	IgM (100%); IgG (96.7%)	IgM (98.8%); IgG (97.5%)	~22 min run	EUA; CE-IVD	[119]
Sienna ™-Clarity COVIBLOCK ™ COVID-19 IgG/IgM Rapid Test Cassette	Clarity Diagnostics, LLC.	WB, EDTA P, SCP, SHP, S	IgM, IgG	IgM (90%); IgG (93.3%)	IgM (100%); IgG (98.8%)	10 min run	EUA; CE-IVD	[120]
Helagen ^®^ COVID-19 IgG/IgM Rapid Test Cassette	Healgen Scientific, LLC.	WB, EDTA P LHP, CSP, S	IgM, IgG	IgM (87.9%); IgG (97.2%)	IgM & IgG (100%)	10 min run	EUA	[121]
SGTI-flux © COVID-19 IgG Test	Sugentech, Inc.	WB, EDTA P, LHP, SCP, SHP, S	IgG	IgG (93.3%)	IgG (100%)	10 min run	EUA; CE-IVD	[122]
**ENZYME-LINKED IMMUNOSORBENT ASSAY (ELISA)**								
COVID-19 Antibody Combo Detection Kit	Symbiotica, Inc.	DBS	IgG	IgG (100%)	IgG (98%)	N/A	EUA; CE-IVD	[123]
COVID-SeroKlir ^®^, Kantaro semi-quantitative SARS-CoV-2 IgG Antibody Kit	Kantaro Biosciences, LLC.	LHP, S	IgG	IgG (98.87%)	IgG (99.6%)	30 min run	EUA	[124]
cPASS ™ SARS-CoV-2 Neutralization Antibody Detection Kit	GenScript USA, Inc.	EDTA P, S	IgG	IgG (100%)	IgG (100%)	15 min run	EUA; CE-IVD	[125]
ZEUS ELISA ™ SARS-CoV-2 IgG Test System	ZEUS Scientific, Inc.	EDTA P, LHP, SCP, S	IgG	93.3%	100%	30 min run	EUA	[126]
COVID-19 ELISA IgG Antibody Test	Mount Sinai Laboratories	EDTA P, S	IgG	92.5%	100%	N/A	EUA	[127]
**CHEMOLUMINESCENT IMMUNOASSY (ChLIA)**								
Dimension EXL SARS-CoV-2 IgG Test	Siemens Healthcare Diagnostics, Inc.	EDTA P, LHP, S	IgG	92%	99.9%	25 min run	EUA	[128]
VITROS ^®^ Immunodiagnostic Products Anti-SARS-CoV-2 Total Reagent Pack	Ortho-Clinical Diagnostics	EDTA P, S	IgM, IgG, IgA	100%	100%	48 min run	EUA	[129]
Elecsys ^®^ Anti-SARS-CoV-2	Roche Molecular Systems	EDTA P, LHP, S	IgM, IgG	88.1%	99.81%	18 min run	EUA	[114,130]
Access ™ SARS-CoV-2 IgM Test	Beckman Coutler	EDTA P, LHP, SCP, S	IgM	95.3%	IgG 100%	30 min run	EUA; CE-IVD	[131]
LIAISON ^®^ SARS-CoV-2 S1/S2 IgG Test	DiaSorin, Inc.	EDTA P, LHP, S	IgG	91.3%	99.8%	30 min run	EUA	[115,132]
BioCheck SARS-CoV-2 IgG Antibody Test Kit	BioCheck, Inc.	S	IgG	100%	100%	30 min run	EUA	[133]
Vibrant COVID-19 Antibody Assay	Vibrant America Clinical Labs	DBS	IgM, IgG	98.1%	98.6%	Home collection: (45 min run in lab)	EUA: CE-IVD	[134]

Abbreviations: PPA, Positive Percentage Agreement; NPA, Negative Percentage Agreement; WB, Whole Blood; P, Plasma (EDTA, Lithium Heparin, Sodium Citrate, Sodium Heparin); S, Serum; DBS, Dried Blood Smear; EUA, Emergency Use Authorization.
